# Extracellular matrix regulation of stress response genes during larval development in *Caenorhabditis elegans*

**DOI:** 10.1093/g3journal/jkac221

**Published:** 2022-08-24

**Authors:** Luke M Chandler, Keith P Choe

**Affiliations:** Department of Biology and Genetics Institute, University of Florida, Gainesville, FL 32611, USA; Department of Biology and Genetics Institute, University of Florida, Gainesville, FL 32611, USA

**Keywords:** *Caenorhabditis elegans*, stress response, extracellular matrix, development

## Abstract

Mutation or loss of 6 extracellular matrix collagen genes disrupts annular furrows in adult *C. elegans* cuticles, causes a wide “Dumpy” body morphology, and activates osmotic, detoxification, and antimicrobial defense genes. High environmental osmolarity reduces internal turgor pressure, physically distorts the epidermis, and activates the same stress responses. Collagen gene mutations that cause Dumpy without furrow disruption do not activate stress responses. These results are consistent with an extracellular damage sensor associated with furrows in the adult cuticle that regulates environmental stress responses in adjacent cells. Several cuticle characteristics change between molts, but all stages have annular furrows and express furrow collagen genes. We compared body shape, furrow organization imaged with differential interference contrast microscopy, and stress response gene expression in furrow collagen gene mutants at all postembryonic stages. We find that most body shape and furrow disorganization phenotypes start at the L3 stage and increase in severity with each molt afterwards. Stress response genes were induced the strongest in adults, correlating with the greatest Dumpy and furrow phenotypes. Although weaker than in adults, osmolyte transporter gene *hmit-1.1* and antimicrobial gene *nlp-29* were also induced in some early larvae that had weak or undetectable cuticle phenotypes. Our data are consistent with progressive cuticle phenotypes in which each new cuticle is at least partially directed by organization of the former cuticle. Gene expression and cuticle data support the role of furrow disruption as a signal in L4 larvae and adults, but also suggest a role for other cuticle organization or epidermal cell effects in early larvae.

## Introduction

Mechanisms that promote homeostasis are essential for animal growth, development, reproduction, and healthy aging. Animals have well-studied and conserved intracellular receptor and signal transduction mechanisms for sensing specific types of environmental stress and activating cytoprotective genes tailored to the condition (Choe 2013; Ermolaeva and Schumacher 2014; Blackwell *et al.* 2015; Urso and Lamitina 2021; Pujol and Ewbank 2022). Epidermal tissues include barrier extracellular matrices (ECMs) that function to limit mechanical damage, penetration of toxins, osmotic pressure gradients, and pathogen infections (Wright 1987; Page and Johnstone 2007; Xu *et al.* 2012; Dodd *et al.* 2018). Barrier ECMs in animals harbor sensory neurons for touch and chemicals that control avoidance and attraction behaviors (Kaplan and Horvitz 1993; Gu *et al.* 1996; Emtage *et al.* 2004; O’Hagan *et al.* 2005). Despite being in direct contact with the environment, little is known about the precise mechanisms by which barrier ECMs sense environmental stress and regulate respective cytoprotective responses in neighboring cells.

A flexible barrier ECM termed the cuticle is a defining characteristic of nematodes (Page and Johnstone 2007). There are over 150 collagen genes in *C. elegans* and a subset of these are secreted from epidermal cells to form complex cuticle structures such as alae and furrows. Genetic screens have identified over 20 collagen genes that cause obvious changes to cuticle and epidermal integrity when mutated or silenced (Cox *et al.* 1980; McMahon *et al.* 2003; Page and Johnstone 2007). Genetic screens identified a few cuticle collagens that are required for regulation of osmotic and innate immune stress responses in the absence of stress (Lamitina 2006; Wheeler and Thomas 2006; Pujol *et al.* 2008; Choe 2013). We systematically screened genetically induced cuticle and epidermal defects for activation of 6 conserved cellular stress responses and discovered that loss of circumferential bands of collagen in the cuticle termed furrows activates osmotic, detoxification, and innate immune responses (Dodd *et al.* 2018). Disruption of other cuticle and epidermal structures did not have the same effects and cytosolic, endoplasmic reticulum, and mitochondria protein misfolding responses were insensitive to furrow disruption (Dodd *et al.* 2018). Loss or mutation of any one of 6collagen encoding genes (*dpy-2*, *3*, *7*, *8*, *9*, and *10)* disrupts furrows and activates stress responses (Dodd *et al.* 2018); DPY-7 and DPY-10 antibodies and DPY-7::GFP fusion protein localize to furrows (McMahon *et al.* 2003; Miao *et al.* 2020). These results and subsequent genetic studies are consistent with a furrow-associated damage sensor that regulates 3 conserved environmental stress responses *via* distinct, but overlapping, downstream signaling (Dodd *et al.* 2018; Wimberly and Choe 2022).

*Caenorhabditis elegans* develops through 5 stages delineated by synthesis and molting of cuticles (Cox and Hirsh 1985; Johnstone 2000). Roles of furrow collagens in stress response regulation have only been studied in the terminal adult stage (Dodd *et al.* 2018; Wimberly and Choe 2022). The cuticle functions as a flexible collagenous barrier and exoskeleton in all stages, but there are differences that could influence environmental stress sensing. *Caenorhabditis elegans* cuticles have distinct basal and cortical layers, and adult is the only stage with a fluid-filled layer sandwiched in the middle (Cox *et al.* 1981). Body shape is more resistant to many collagen mutations in early larval stages than in adults (Cox *et al.* 1980). Cuticle thickness increases with each molt and on the surface of the cortical layer, longitudinal ridges termed alae are only present in L1 larvae and adults (Cox *et al.* 1981); furrows are present in all stages and all molts are preceded by a burst in expression of furrow collagen genes *dpy-2, 3, 7, 8, 9*, and *10* (McMahon *et al.* 2003). The presence of furrows and expression of furrow collagens in all stages raises the possibility that the damage sensor functions throughout larval development. Alternatively, changes in cuticle composition and downstream signaling could cause variation in sensor function.

We compared body shape, furrow organization, and stress response gene activation between developmental stages in 3 furrow collagen gene mutants. Body shape became progressively more Dumpy (wide and short) in mutants during development starting at the L2 or L3 stage. Using high magnification differential interference contrast (DIC) imaging, we observed well-organized furrows in wild-type worms at all stages. Furrows were well-organized in L1 and L2 larvae of all 3 mutants but became progressively less-organized with each stage starting at L3. Canonical osmolyte synthesis gene *gpdh-1*, osmolyte transporter gene *hmit-1.1*, and antimicrobial gene *nlp-29* were all induced the strongest in adults of furrow collagen gene mutants correlating with severity of body shape and furrow phenotypes. Surprisingly, there were also cases where *hmit-1.1* and *nlp-29* were induced in early larval stages that had wild-type body shape and furrow organization; this result suggests that these genes may also respond to other cuticle or epidermal cell phenotypes. Lastly, a functional DPY-7::GFP fusion protein formed furrows in all stages of wild-type worms but was not a reliable marker for furrows in *dpy-3* worms.

## Materials and methods

### *Caenorhabditis elegans* strains and culture conditions

All *C. elegans* strains were cultured at 20°C using standard methods (Brenner 1974) The following strains were used: N2 Bristol, CB88 *dpy-7(e88)* X, CB12 *dpy-9(e12)* IV, QV365 *dpy-3(ok2263)* X, XW18042 q*xls722* [*dpy-7p::dpy-7::sfGFP*] II, QV359 *dpy-3(ok2263)* X; *qxls722* II, and QV360 *dpy-9* IV; *qxls722* II.

Worms were synchronized by bleach and early larval stages were collected at times previously established for 20°C (Corsi *et al.* 2021). L4-staged worms were identified by a clear spot at the developing vulva. Young adults were identified by a fully developed vulva before development of embryos.

### qRT-PCR

qRT-PCR assays were run using the delta-delta Ct method with primer efficiencies determined from standard curves as described previously (Scolaro *et al.* 2019) with the following modifications; after lysis, genomic DNA was removed with dsDNAse (Thermo Fisher product EN007). Numbers of worms picked to each replicate were adjusted to account for worm sizes as follows: 20 L1, 15 L2, or 10 L3 larvae for all strains; 8 wild-type or 12 *dpy* L4 larvae; 7 wild-type and 10 *dpy* young adults. All qRT-PCR reactions were performed in 10 μL reaction volumes in a Realplex^2^ (Eppendorf AG, Hamburg, Germany). Relative expression was normalized to a value of 1.0 for wild-type worms on standard NGM agar within each developmental stage using an average of 3 reference genes (*rpl-2*, *tba-1*, and *cdc-42*). Primers used for qRT-PCR are listed in Supplementary Table 1.

### Microscopy

Images of whole worms were taken with a Zeiss Discovery V12 Stereo microscope and OptixCam Summit camera; body shape was calculated as a ratio of average width to total length using ImageJ version 1.53r (Schindelin *et al.* 2012). Lengths were measured as a free-form line from head to tail along the center of worms. Width of each worm was an average of 3 lines perpendicular to the long axis: 1 at the midpoint, 1 at the junction of the pharynx and intestine, and 1 half-way between the midpoint and rectum.

Furrows were imaged by DIC on an Olympus BX60 microscope with an oil 60× UPlanFl objective (numerical aperture at 1.25) and additional 2× lens in front of a Zeiss Axiocam MRm camera (Thornwood, NY). Density of furrows was counted manually along 10–60 µm regions in Image J. DPY-7::GFP was imaged with the same objective, but without the additional 2× lens; focal plane was consistent between paired DIC and fluorescent images at either the cuticle or epidermis. Contrast and brightness adjustments to fluorescence images were made evenly in whole images and equally between strains at the same developmental stage.

### Statistical analysis

All means were compared to control with unpaired *t*-tests with Welch’s corrections (Ruxton 2006). *P*-values were corrected for false-discovery rate with the Benjamini–Hochberg method. Log base 2 of relative mRNA levels was used to calculate *P*-values for gene expression comparisons. Categorical data were compared with Fisher’s exact tests in Prism 5.04 (La Jolla, CA). Data were graphed with Prism 5.04.

## Results and conclusions

### Cuticles are more resistant to furrow collagen gene mutations in early larvae than adults

**Fig. 1.**
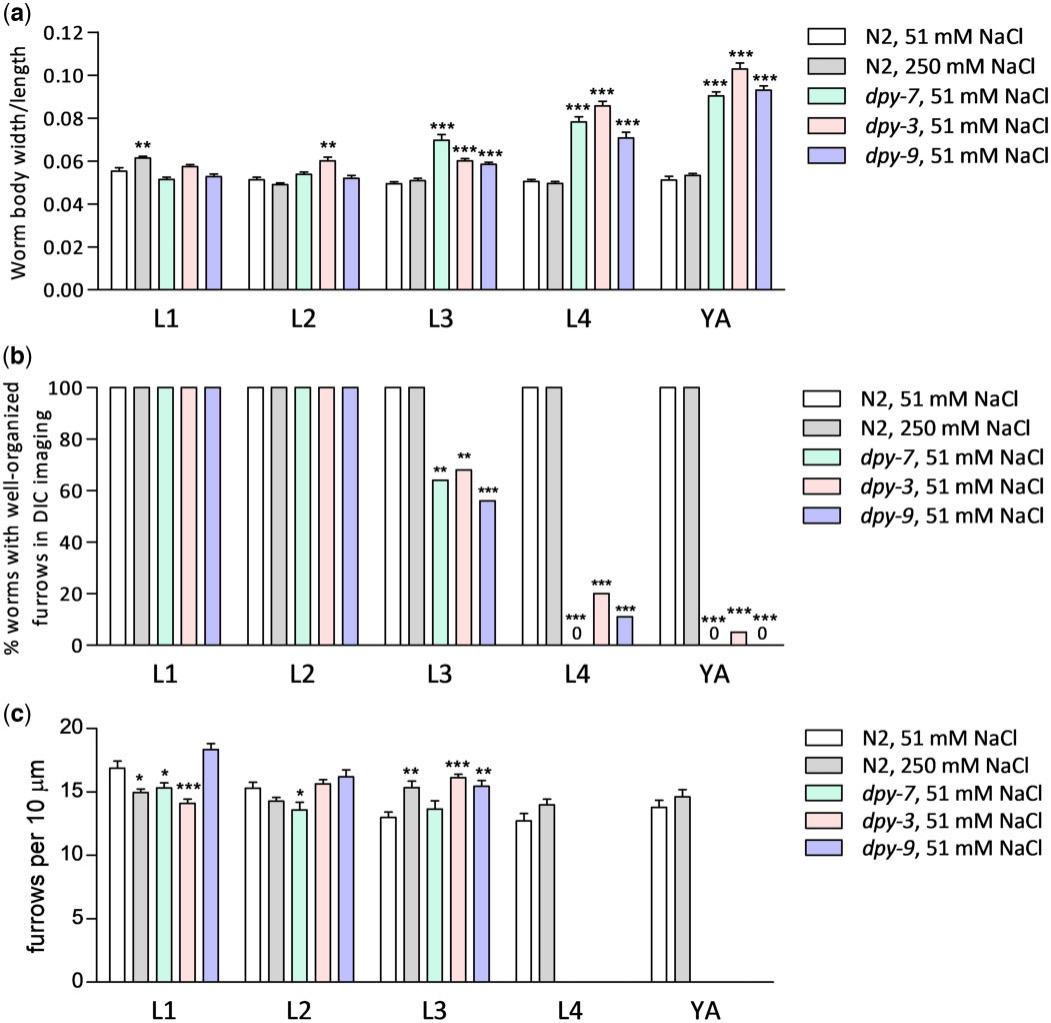
Cuticles are more resistant to furrow collagen gene mutations in early larvae than adults. a) Mean width/length ratios at each developmental stage plus SE; *N* = 10–18 individual worms. b) Percentage of worms with clearly organized furrows visible with high magnification DIC at each developmental stage, *N* = 10–39 worms. c) Mean density of furrows per 10 µm plus SE, *N* = 10–23 individual worms; furrows were not organized enough to measure density for *dpy* worms at L4 or young adult (YA) stages. *P *<* 0.05, **P *<* 0.01, and ***P *<* 0.001 vs N2 worms on 51 mM NaCl.

There are over 20 genes that cause a short and wide “Dumpy” body morphology in adults when mutated; we previously demonstrated that loss of any one of only 6 of these (*dpy-2, 3, 7, 8, 9*, and *10*) disrupts furrows and activates osmotic, detoxification, and innate immune response genes in adults (Dodd *et al.* 2018). Here, we used *dpy-7(e88)*, *dpy-9(e12)*, and *dpy-3(ok2263)* alleles; *dpy-7(e88)* and *dpy-9(e12)* are missense mutations and *dpy-3(ok2263)* is the only deletion allele of a furrow collagen gene that is publicly available. As shown in Fig. 1a, adults of all 3 mutants are Dumpy (i.e. larger width/length ratios) as expected (Cox *et al.* 1980). There was no effect of *dpy-7(e88)* or *dpy-9(e12)* on body shape until the L3 stage and effects worsened with each molt afterwards (Fig. 1a); *dpy-3(ok2263)* was wider than wild type in all stages except for L1 with progressively larger effects in L4 and adult stages. These quantitative results confirm prior categorical data demonstrating that body shape becomes more sensitive to collagen mutations with each molt (Cox *et al.* 1981).

We also grew wild-type N2 worms on agar containing 250 mM NaCl starting at the egg stage; high salt levels activate osmotic, innate immune, and detoxification responses in adults and is likely an environmental condition partially mimicked by furrow loss (Lamitina *et al.* 2006; Pujol *et al.* 2008; Choe 2013; Dodd *et al.* 2018). As shown in Fig. 1a, body shape was not affected by chronic exposure to 250 mM NaCl except for a small effect at L1.

**Fig. 2.**
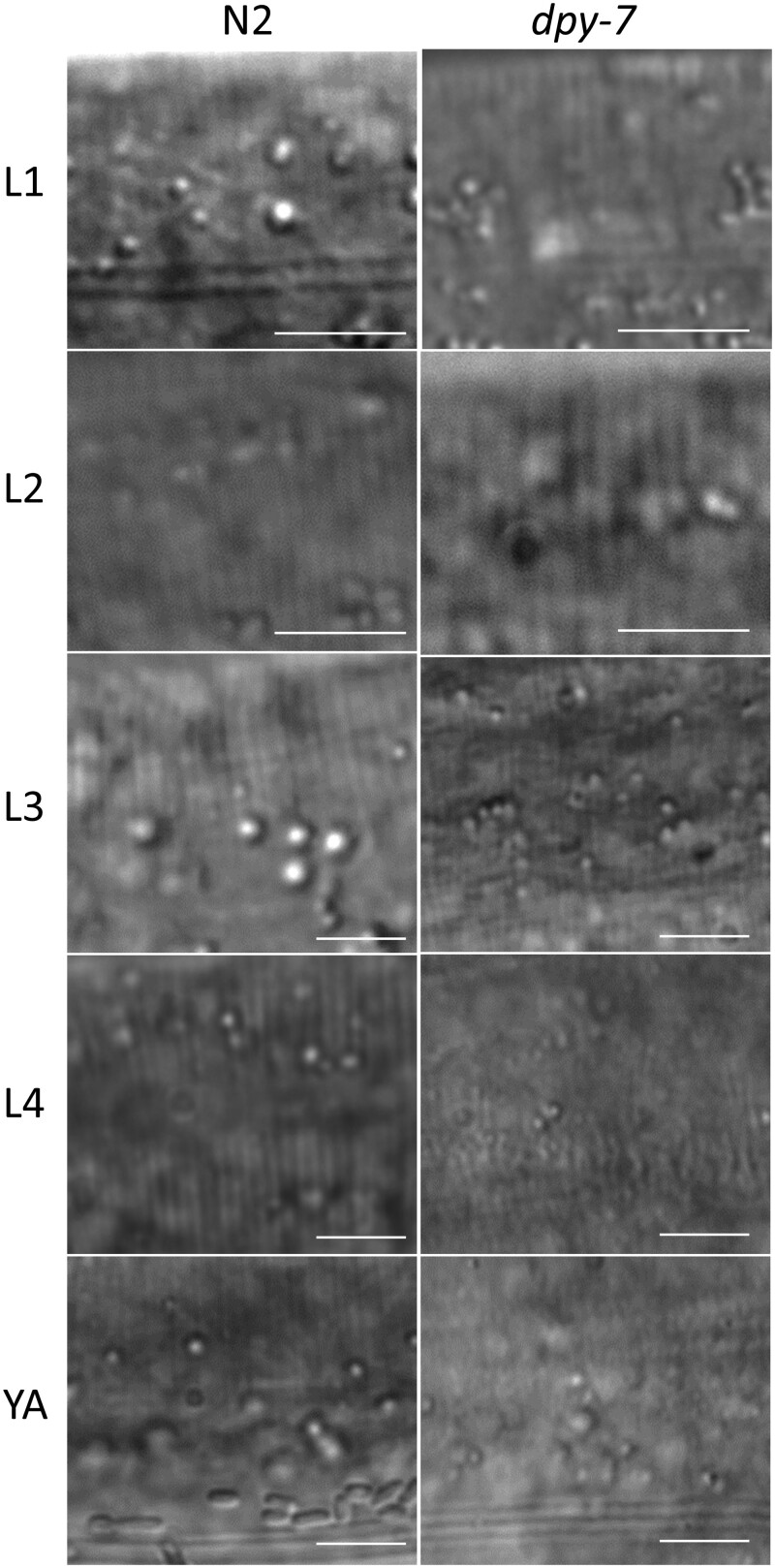
Furrow organization is disrupted in L4 and adult *dpy-7* worms. Representative micrographs of cuticles in N2 and *dpy-7* worms taken at 120× magnification. Scale bars are 5 µm.

We next compared severity of cuticle furrow defects between developmental stages. We and others previously used a COL-19::GFP fusion protein to visualize cuticle structure, but COL-19 is adult specific (Thein *et al.* 2003; Dodd *et al.* 2018). Here, we used DIC microscopy at 120× magnification to visualize and score furrow organization (Figs. 1, b and c and 2; Supplementary Fig. 1). Figure 1b shows categorical data (organized or disorganized) for furrows by stage and condition. We observed organized furrows in all wild-type worms at all stages including under chronic exposure to 250 mM NaCl. All collagen mutant worms also had organized furrows at L1 and L2. At L3, 56–68% of collagen mutant worms had well-organized furrows visible with DIC; this percentage decreased to 0–20% at L4 and only 0–5% in adults (Figs. 1b and 2; Supplementary Fig. 1).

In the first 3 larval stages where enough images of organized furrows in all conditions could be scored, furrow densities were compared to wild type. Unlike body shape and furrow organization (Fig. 1, a and b), furrow density effects were weak and inconsistent between mutants (Fig. 1c).

### Stress response gene sensitivity to furrow collagen gene mutation varies during development

**Fig. 3.**
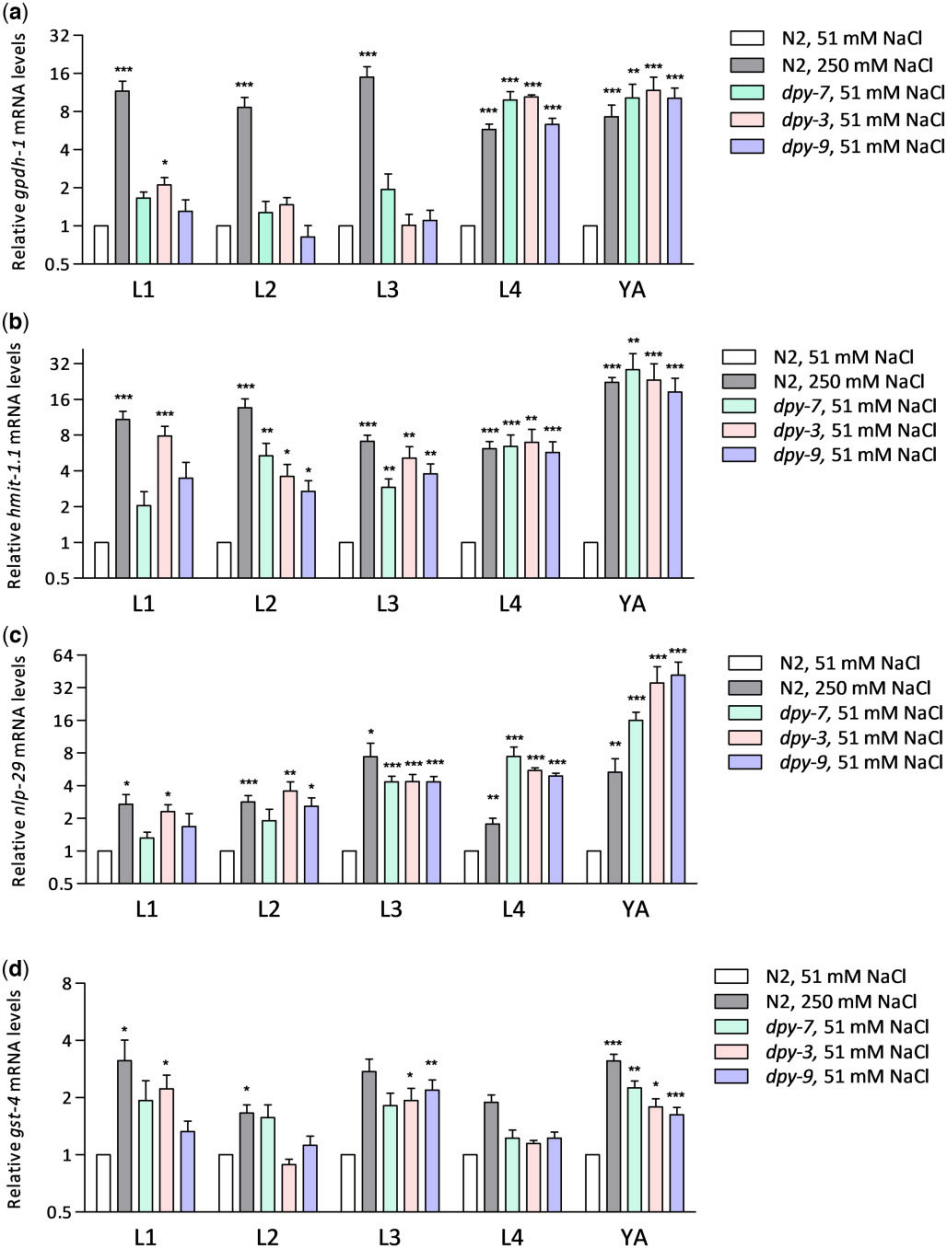
Mean relative mRNA levels plus SE for a) *gpdh-1*, b) *hmit-1.1*, c) *nlp-29*, and d) *gst-4* in all conditions measured at all developmental stages. Values are normalized to 1.0 for N2 worms grown at 51 mM NaCl. *N* = 4–12 samples of 6–15 worms per replicate. *P *<* 0.05, **P *<* 0.01, and ***P *<* 0.001 vs N2 at the same stage using Welch’s *t*-tests for uneven variances and Benjamini–Hochberg corrections on log base 2 relative expression values.

We and others have established canonical osmotic *(gpdh-1, hmit-1.1)*, detoxification (*gst-4)*, and innate immune (*nlp-29)* response genes that are activated by furrow loss in adults (Lamitina *et al.* 2004; Lamitina *et al.* 2006; Pujol *et al.* 2008; Choe 2013; Dodd *et al.* 2018); *gpdh-1* and *hmit-1.1* encode an enzyme and transporter responsible for osmolyte accumulation, respectively, *gst-4* encodes a detoxification enzyme and is a direct target of SKN-1/Nrf, and *nlp-29* encodes an antimicrobial peptide (Lamitina *et al.* 2004; Kahn *et al.* 2008; Pujol *et al.* 2008; Choe *et al.* 2009; Kage-Nakadai *et al.* 2011). To compare stress response gene activation between developmental stages, we used RT-qPCR and normalized to N2 wild-type worms within each stage. As shown in Fig. 3, all 4 stress response genes were induced in adult furrow collagen gene mutants as expected. Chronic exposure to 250 mM NaCl significantly activated *gpdh-1*, *hmit-1.1*, and *nlp-29* in all developmental stages (Fig. 3) indicating that all stages are capable of inducing these genes in response to an environmental stimulus; *gst-4* induction was statistically significant at all stages except L3 and L4 (Fig. 3d).

Expression of *gpdh-1* was only strongly induced by furrow mutations in L4 and adult stages (Fig. 3a), which is consistent with complete or nearly complete furrow disorganization (Figs. 1b and 2; Supplementary Fig. 1). There was no *gpdh-1* induction in *dpy* mutants at L3 despite partial furrow disorganization (32–44%) suggesting that there may be granular levels of furrow organization relevant to stress response signaling that are not easily recognized with DIC microscopy.

Other stress responsive mRNAs had different patterns than *gpdh-1* in early larvae. *hmit-1.1* and *nlp-29* mRNAs were induced as early as L1 in *dpy-3* mutants with a general trend of increasing induction with each molt for all 3 mutants (Fig. 3, b and c). The greatest induction of *hmit-1.1* and *nlp-29* in adults correlates with the greatest furrow and body shape phenotypes (Figs. 1, a and b and 2). Alternatively, induction in L1 and L2 worms that lack furrow organization and body shape phenotypes suggests sensitivity to other phenomenon resulting from collagen mutation or changes to furrows that are more subtle than we can observe with DIC. Effects on *gst-4* mRNA were weak compared to the other genes and induction was the most consistent in adults.

### DPY-::GFP localization during development

**Fig. 4.**
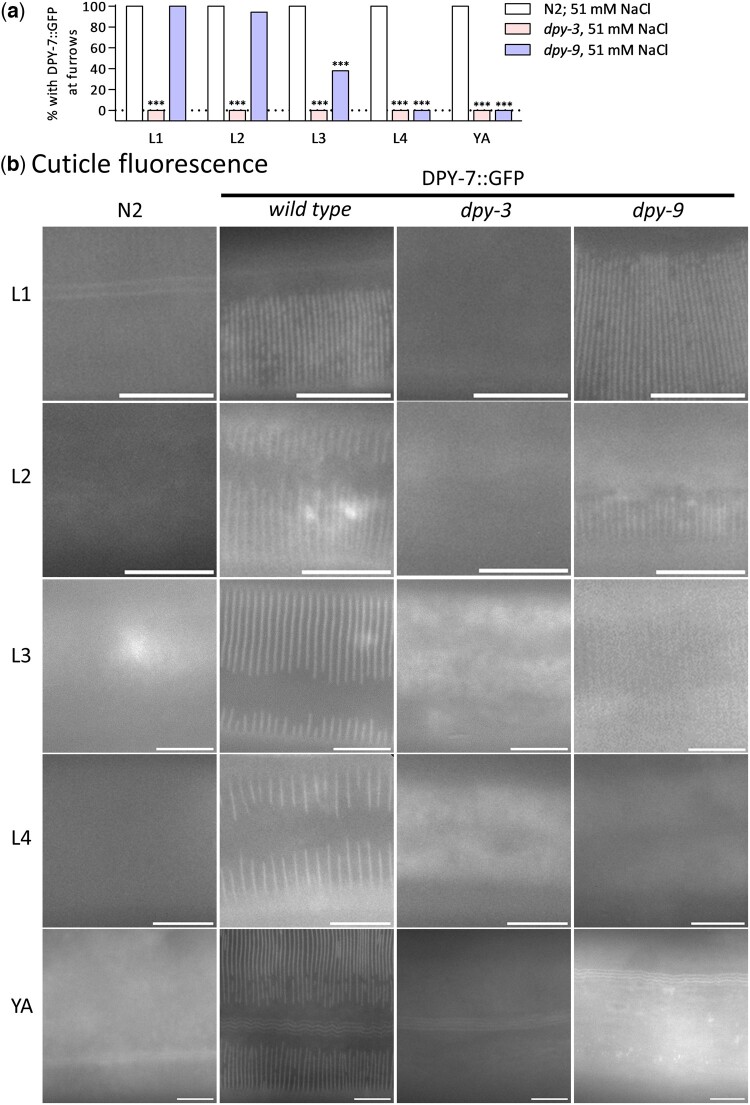
Localization of DPY-7::GFP. a) Percentage of worms DPY-7::GFP localized to furrows in N2, *dpy-3*, and *dpy-9* worms. b) Representative fluorescence images at all postembryonic stages. Images of the same worms with DIC are in Supplementary Fig. 2. Scale bars are 10 µm.

We also imaged a functional DPY-7::GFP fusion protein as a potential marker for cuticle organization in larvae and to investigate interactions between furrow collagens (Miao *et al.* 2020). Because DPY-7::GFP rescues *dpy-7(e88)*, we were limited to testing *dpy-9(e12)* and *dpy-3(ok2263).* As shown in Fig. 4, DPY-7::GFP localizes to furrows at all developmental stages of wild-type worms. As expected based on prior antibody labeling (McMahon *et al.* 2003), DPY-7::GFP localization to furrows was absent from *dpy-9(e12)* and *dpy-3(ok2263)* adults (Fig. 4, a and b and Supplementary Fig. 2). In *dpy-9* worms, DPY-7::GFP localization to furrows closely correlated with furrow organization scored with DIC during development (Figs. 1b and 4). Alternatively, DPY-7::GFP was absent from furrows in all stages in *dpy-3(ok2263)* deletion worms (Fig. 4, a and b and Supplementary Fig. 2). These results suggest that furrows can be formed in early larvae without including DPY-7. In some stages that lacked localization to furrows, DPY-7::GFP fluorescence was diffuse throughout epidermal cells (Supplementary Fig. 3).

## Discussion

### Cuticle sensitivity to furrow collagen gene mutations increases during development

Data in Fig. 1 quantitatively confirm that *C. elegans* body shape becomes more sensitive to furrow collagen gene mutations with each molt (Cox *et al.* 1980); it also demonstrates that furrow organization becomes progressively more disrupted with each molt starting at L3. Furrow collagens and furrows are present in every stage of wild-type worms (McMahon *et al.* 2003), but it is possible that variation in other collagens and cuticle components may contribute to the developmental variation seen in the mutants (Cox *et al.* 1980). The progressive increase in cuticle defects may be caused by a cumulative mechanism in which collagen organization in each new cuticle is at least partially directed by the previous cuticle. Roller, another phenotype caused by collagen gene mutations, also increases in severity during development, consistent with a cumulative mechanism (Cox *et al.* 1980).

### Responses to furrow collagen gene mutation increase during development

In adults, induction of osmotic, detoxification, and antimicrobial response genes is caused by collagen gene mutations that disrupt furrows and cause Dumpy body shape; collagen gene mutations that cause only Dumpy are not sufficient to induce stress response genes, supporting a model in which furrow disruption is sensed as environmental stress (Dodd *et al.* 2018). In our current study, the stress response genes measured were most sensitive to furrow collagen gene mutations in adults that have the greatest changes in body shape and furrow organization.

Quantitative cuticle phenotypes and stress response gene expression in larval stages reveal unexpected complexity. Although weaker than in adults, *hmit-1.1* and *nlp-29* were induced in early larval stages of furrow collagen gene mutants that had organized furrows and little to no change in body shape (Figs. 1–3). These results suggest that the damage sensor is sensitive to more subtle furrow changes than previously appreciated or that other aspects of the cuticle or epidermal cells are acting as a signal in early larvae. Unfortunately, DPY-7::GFP was an unreliable marker of furrow organization in *dpy-3* early larvae (Figs. 1, 2, and 4). In *dpy-9* worms, DPY-7::GFP localization to furrows in L1 and L2 larvae was similar to wild type consistent with a nonfurrow signal activating *hmit-1.1* and *nlp-29*.

The striking difference between *hmit-1.1* and *gpdh-1* expression in early furrow collagen gene mutant larvae is surprising because they both respond strongly to hyperosmotic stress and function in osmolyte accumulation (Kage-Nakadai *et al.* 2011; Lamitina *et al.* 2004). Both responded strongly to high osmolarity throughout development demonstrating that both are capable of being activated at any stage (Fig. 3). These results are consistent with *hmit-1.1* sensitivity to an alternative signal in early larvae. Genetic screens for *hmit-1.1* regulators similar to those previously conducted for *gpdh-1* (Lamitina *et al.* 2006; Urso and Lamitina 2021) may therefore identify new mechanisms of osmotic signal transduction.

## Supplementary Material

jkac221_Supplemental_Legends

jkac221_Table_S1

jkac221_Supplementary_Figure_S1

jkac221_Supplementary_Figure_S2

jkac221_Supplementary_Figure_S3

## Data Availability

Strains are available on request. Underlying numeric data are posted at https://doi.org/10.6084/m9.figshare.18515420.v3. Supplemental material is available at G3 online.
