# Supplementary material for: Extracellular matrix regulation of stress response genes during larval development in Caenorhabditis elegans
Source: G3 (Bethesda). 2022 Aug 24;12(11):jkac221. doi: 10.1093/g3journal/jkac221 (PMC9635657; doi:10.1093/g3journal/jkac221)
Supplement: jkac221_Supplemental_Legends [file jkac221_supplemental_legends.docx]

**Supporting Legends**

Table 1. Primers used are listed.

Figure S1. Furrow organization is disrupted in L4 and adult *dpy-7, dpy-3,* and *dpy-9* worms. Representative DIC micrographs of cuticles taken at 120X magnification. Scale bars are 5 µm.

Figure S2. Localization of DPY-7::GFP. DIC micrographs taken at the same focal planes and fields of view as Fig. 4B.

Figure S3. DPY-7::GFP localizes to hypodermal cells in *dpy-3* and *dpy-9* larvae. Paired DPY-7::GFP fluorescence and DIC micrographs focused at epidermal cells. Scale bars are 10 µm.
