# Supplementary figures and images for: Extracellular matrix regulation of stress response genes during larval development in Caenorhabditis elegans
Source: G3 (Bethesda). 2022 Aug 24;12(11):jkac221. doi: 10.1093/g3journal/jkac221 (PMC9635657; doi:10.1093/g3journal/jkac221)

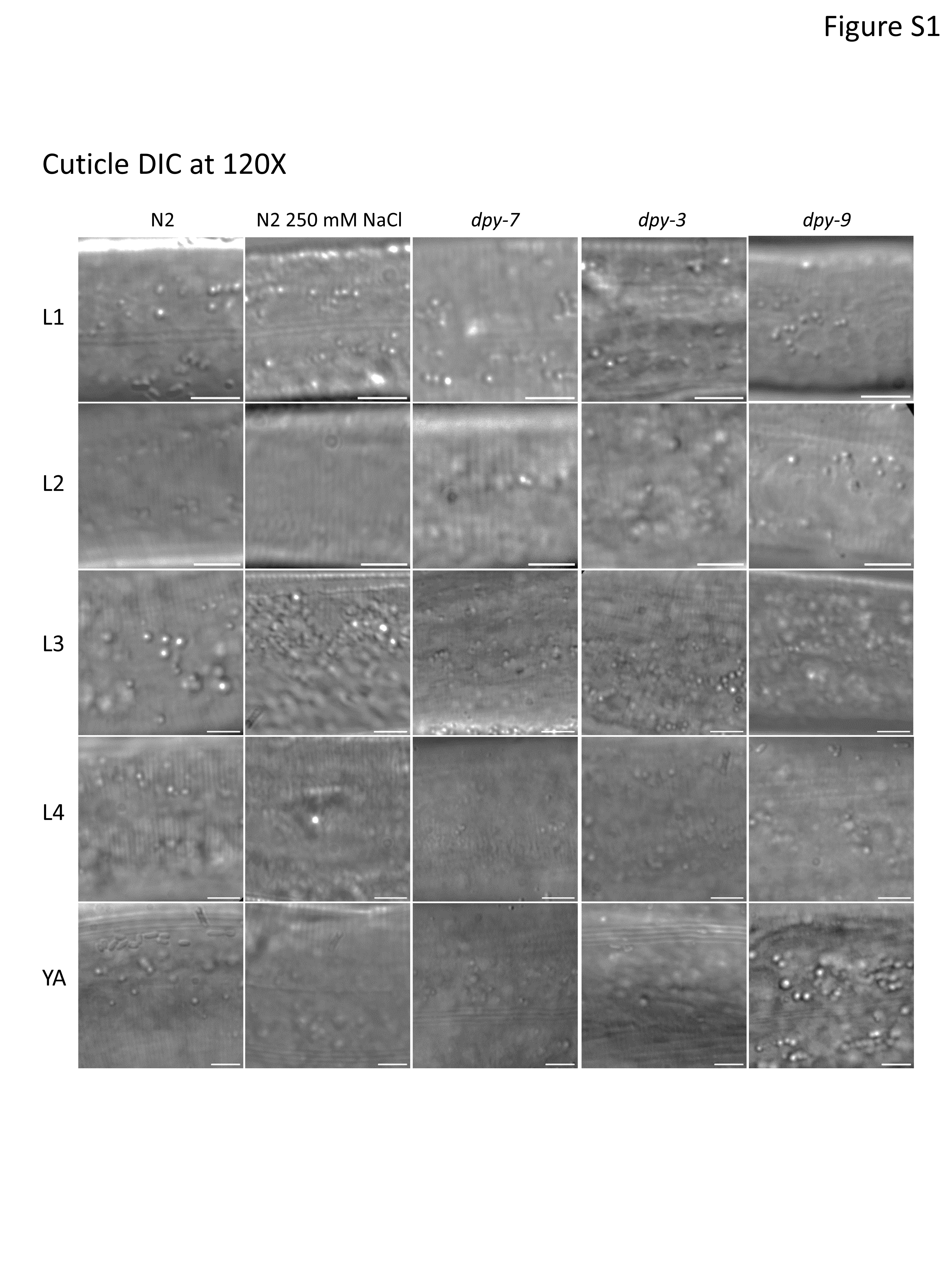

Supplement: jkac221_Supplementary_Figure_S1 [file jkac221_supplementary_figure_s1.jpeg]

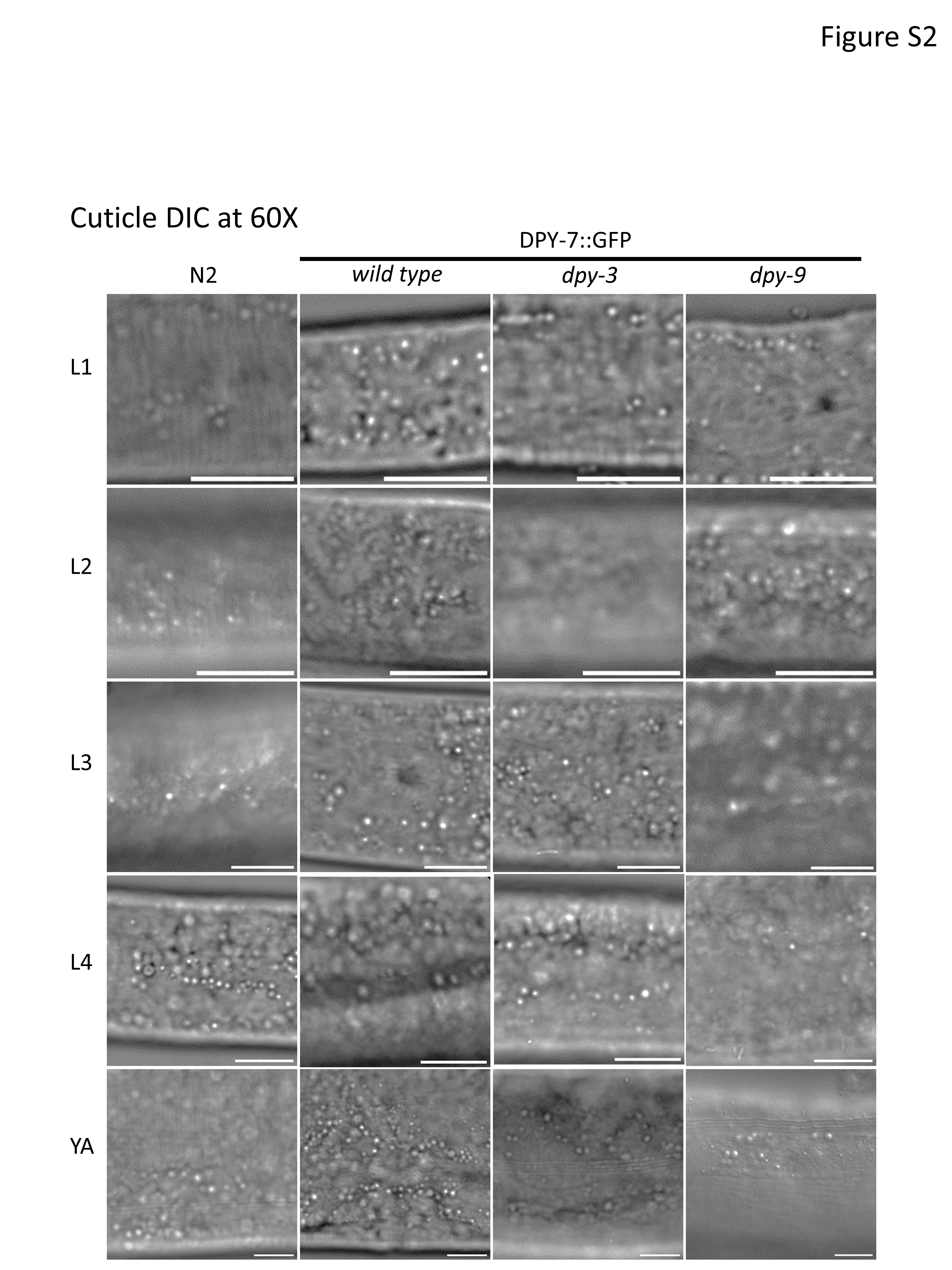

Supplement: jkac221_Supplementary_Figure_S2 [file jkac221_supplementary_figure_s2.jpeg]

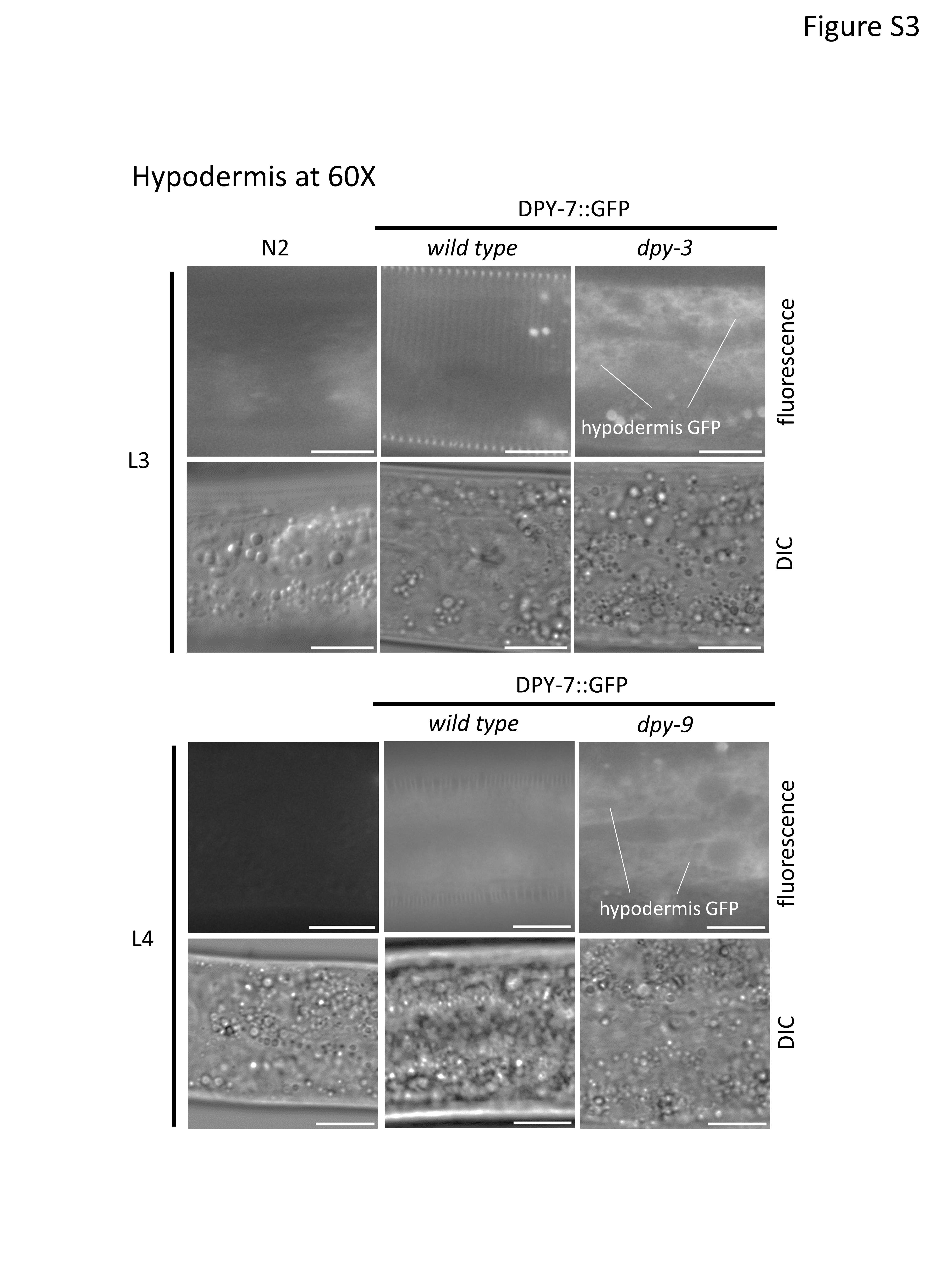

Supplement: jkac221_Supplementary_Figure_S3 [file jkac221_supplementary_figure_s3.jpeg]
